# The influence of COMT Val^158^Met genotype on the character dimension cooperativeness in healthy females

**DOI:** 10.1002/brb3.233

**Published:** 2014-04-21

**Authors:** Chris Baeken, Stephan Claes, Rudi De Raedt

**Affiliations:** 1 Department of Psychiatry and Medical Psychology, Ghent UniversityGhent, Belgium; 2 Department of Psychiatry, University Hospital (UZBrussel)Brussels, Belgium; 3 Ghent Experimental Psychiatry (GHEP) LabGhent, Belgium; 4 University Psychiatric Center KU LeuvenLeuven, Belgium; 5 Department of Experimental Clinical and Health Psychology, Ghent UniversityGhent, Belgium

**Keywords:** Catechol-O-methyltransferase, cooperativeness, personality

## Abstract

**Objectives:**

Although the Val^158^Met catechol-O-methyltransferase (COMT) gene has been linked with the temperament dimension Novelty Seeking (NS), new insights in this polymorphism might point to a major role for character features as well. Given that individual life experiences may influence Val^158^ and Met^158^ allele carriers differently it has been suggested that the character trait cooperativeness could be implicated.

**Case report:**

A homogeneous group of eighty right-handed Caucasian healthy female university students were assessed with the TCI and genotyped for the COMT Val^158^Met polymorphism (rs4680). Gene determination showed that eighteen were Val^158^ homozygotes, forty-four Val/Met^158^ heterozygotes, and eighteen were Met^158^ homozygotes. All were within the same age range and never documented to have suffered from any neuropsychiatric illness. Bonferroni corrected non-parametric analyses showed that only for the character scale cooperativeness Val^158^ homozygotes displayed significant higher scores when compared to Met^158^ homozygotes. No significant differences on cooperativeness scores were found between Val^158^ and Val/Met^158^ carriers or between Met^158^ and Val/Met^158^ carriers. No differences were observed for the COMT Val^158^Met polymorphism and the other temperament and character scales.

**Conclusions:**

Our findings support the assumption that the Val^158^Met single nucleotide polymorphism (SNP) influences character traits and not only temperament. Our results add to the notion that Val^158^ homozygotes are considered to be helpful and empathic and it suggest that these cooperativeness character traits are related to the dopaminergic system.

## Introduction

Notwithstanding that the concept of “personality” has been thoroughly investigated, the interplay between personality features and genetics remains poorly understood. The Val^158^Met polymorphism of the catechol-O-methyltransferase (COMT) gene (rs4680), which is involved in the degradation of the dopamine neurotransmitter, has been associated with personality (disorders) and with a range of psychiatric illnesses (Hosák 2007; Calati et al. 2011; Witte and Flöel 2012). Although Val^158^ carriers may display more resilience (Kang et al. 2013), Met^158^ allele carriers have been commonly related with a higher risk for emotional dysregulation (Kempton et al. 2009). However, the current literature is not consistent on this issue. Some studies reported that the Met^158^ allele is associated with increased limbic responsiveness to negative stimuli (Smolka et al. 2005, 2007), whereas others reported the opposite (Kempton et al. 2009; Domschke et al. 2012) or described null results (Drabant et al. 2006). In Cloningers' influential psychobiological model of personality (Cloninger et al. 1994), the dopaminergic-related temperament dimension Novelty Seeking (NS), and to some extent the serotonergic-associated temperament Harm Avoidance (HA), have been linked with the COMT Val^158^Met gene; the latter, however, mostly found in Asian populations (Montag et al. 2012).

Interestingly, Baumann et al. (2013) found that early aversive life experiences might increase the vulnerability toward anxiety disorders in COMT Met^158^ allele carriers and Drury et al. (2010) showed that early severe social deprivation was associated with a higher risk to develop major depression disorder in Val^158^ homozygotes. These findings suggest that not only genetically heritability dimensions such as temperament can be modulated by the COMT Val^158^Met gene but also by environmental factors. Indeed, whereas “Temperament” refers to biases in automatic responses to emotional stimuli and is to some extent independently heritable, “Character” refers to individual differences in self-object relationships, which develop in a stage-like manner as a result of nonlinear interactions among temperament, family environment, and individual life experiences (Cloninger et al. 1994).

Pełka-Wysiecka et al. (2012) found that the dopamine transporter (DAT) gene was positively correlated with the character scale cooperativeness (CO: helpful and empathic vs. hostile and aggressive) in women without psychiatric disorders. Moreover, Caucasian carriers of at least one Val^158^ allele showed a greater effect for social facilitation and cooperativeness (working together in group) than Met^158^ homozygotes (Walter et al. 2011). In an experimental study testing COMT Val^158^Met polymorphism for altruistic behavior, Reuter et al. (2011) found that the highest correlation between the amounts of donations was observed for CO. Although the Val^158^ allele was related to the level of altruism, CO was not. Because no effects of gender were examined this could explain to some extent the lack of such association. Besides age also gender may confound COMT Val^158^Met gene results (Harrison & Tunbridge 2008), also related to personality (Chen et al. 2011).

Consequently, we hypothesized that in a homogeneous sample of Caucasian females, selected within a narrow age range, never documented to have suffered from any neuropsychiatric illness, that individual scores on the temperament dimension NS and the character scale CO would differ for COMT Val^158^ and Met^158^ allele carriers. We expected that Val^158^ allele carriers would display lower scores on NS and higher scores on CO. We did not expect any interaction with the other scales of Cloningers' Temperament and Character Inventory (TCI; Cloninger et al. 1994).

**Figure 1:**
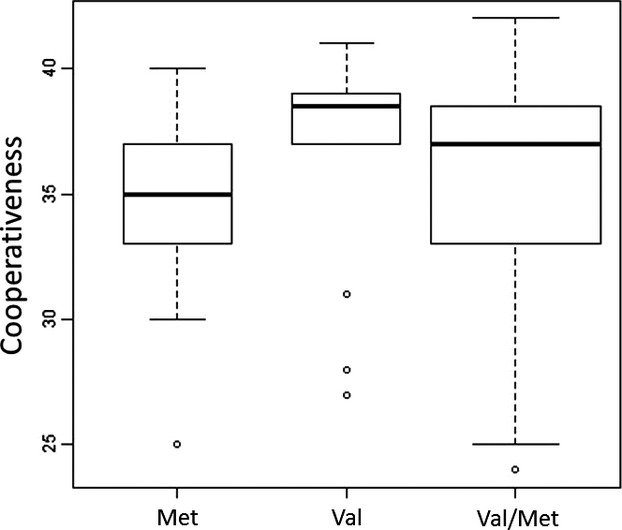
Boxplot representation of the COMTVal^158^Met gene in relation to the individual scores on cooperativeness (*y*-axis).

## Material and Methods

### Participants

The study was approved by the ethics committee of our University Hospital (UZBrussel) and all subjects gave written informed consent. Eighty right-handed Caucasian female participants, all university students, were recruited (mean age = 21.7 years, SD = 2.5). Right-handedness was assessed with the van Strien questionnaire (Van Strien and Van Beek 2000). None had ever used major psychotropic medications and all were free of any drug. Subjects taking medication, other than birth-control pills, were excluded. To exclude psychiatric or neurological diseases all volunteers were screened by the first author (C.B.). Psychiatric disorders were assessed by the Dutch version of the Mini-International Neuropsychiatric Interview (MINI) (Sheehan et al. 1998). Subjects with a psychiatric disorder and/or a score higher than 8 on the Beck Depression Inventory (BDI-II; Beck et al. 1996) were excluded. All were assessed using a Dutch version of the Temperament and Character Inventory (TCI) (de la Rie et al. 1998).

### Temperament and character inventory

The Temperament and Character Inventory (TCI) is a 240-item questionnaire developed by Cloninger (1987) and Cloninger et al. (1994). The TCI consists of four temperament scales [Harm Avoidance (HA), Novelty seeking (NS), Reward dependence (RD), Persistence (P)], and three character scales [Cooperativeness (CO), Self-directedness (SD), and Self Transcendence (ST)].

### Genetics

In a first step, EDTA acid anti-coagulated blood samples were drawn from each participant and DNA was isolated. Second, genotyping of COMT rs4680 single-nucleotide polymorphism (SNP) was performed using the MassARRAY platform (SEQUENOM, San Diego, CA).

### Statistical analysis

All collected data were analyzed with SPSS 22 (Statistical Package for the Social Sciences; IBM SPSS Statistics for Windows, Version 22.0, IBM Corp., Armonk, NY). The significance level was set at *P* ≤ 0.05, two-tailed.

The Shapiro–Wilk normality test showed that the temperament and character scale scores were not normally distributed (*P'*s < 0.05). Log transformation or square root transformation did not result in normality. Therefore, nonparametric Kruskal–Wallis and Mann–Whitney *U* test analyses were used. We used each of the seven scales of Cloningers' Temperament and Character Inventory as dependent variable in separate analyses. The independent variables in the follow-up Mann–Whitney *U* test were the three genotypes (Val^158^, Val^158^Met, Met^158^). Follow-up Mann–Whitney *U* tests were Bonferroni corrected for the number of significant main effects and contrasts within these main effects.

## Results

From the 80 participants, 18 were Val^158^ homozygotes, 44 Val/Met^158^ heterozygotes, and 18 were Met^158^ homozygotes. See also Table 1 and Figure 1. The calculation of the Hardy–Weinberg Equilibrium for two alleles showed no deviation of this assumption (*χ*^2^[1, *N* = 80] = 0.80, *P* = 0.37).

**Table 1:** Group variables catechol-O-methyltransferase (COMT) Val^158^Met and temperament and character inventory (TCI) scores: medians and ranges

			TCI						
			
Group	Number	Age	NS	HA	RD	P	SD	CO	ST
All	80	21 (12)	22 (26)	15 (29)	19 (15)	4 (8)	34 (33)	37 (18)	7 (27)
Val^158^	18	22.5 (11)	22.5 (25)	14.5 (22)	18 (13)	5 (8)	34.5 (26)	38.5 (14)	6.5 (21)
Val/Met^158^	44	21 (10)	22.5 (25)	15.5 (28)	19 (14)	3.5 (8)	33 (32)	37 (18)	7 (27)
Met^158^	18	21 (7)	21 (21)	14.5 (27)	18.5 (15)	4.5 (6)	34.5 (26)	35.0 (15)	9 (17)

The results of the Kruskal–Wallis test indicate that there is a significant difference in the medians of Val^158^ – Val/Met^158^ – Met^158^ allele carriers, however, this was only for the temperament scale Persistence (*χ*^2^[2, *N* = 80] = 6.24, *P* = 0.04) and the character scale Cooperativeness (*χ*^2^[2, *N* = 80] = 8.24, *P* = 0.02). All other TCI scales were not significant (*P*'s > 0.05).

To follow-up on both significant main effects, Mann–Whitney *U* test revealed that for the character scale Cooperativeness Val^158^ homozygotes displayed significant higher CO scores when compared to Met^158^ homozygotes (*z* = −2.84, *n* – ties = 36, *P* = 0.03). No significant differences on CO scores were found between Val^158^ and Val/Met^158^ carriers (*z* = −1.75, *n* – ties = 62, *P =* 0.48) or between Met^158^ and Val/Met^158^ carriers (*z* = −1.68, *n* – ties = 62, *P* = 0.55). Mann–Whitney *U* test revealed no significant group differences for the temperament scale Persistence (*P*'s > 0.05). These Mann–Whitney *U* tests were Bonferroni corrected for the six comparisons.

Finally, the Kruskal–Wallis test did not show differences in age between the three groups (*χ*^2^[2, *N* = 80] = 4.07, *P* = 0.13).

## Discussion

In contrast to our initial hypothesis, we did not observe an influence of the COMT Val^158^Met polymorphism on the temperament dimension NS. Only one study observed that female Met^158^ carriers show higher NS scores (Golimbet et al. 2007). As to how this differs from our study is not easy to explain as the sample size was similar to ours (*n* = 74). One could speculate that the choice of their participants, all born in Moscow, Russia, with a wider age range and the fact that the authors used a shortened TCI version (with 125 instead of 204 items) could account for some of the discrepancies. Furthermore, other studies showing an association with NS and the COMT Val^158^Met gene observed such phenomena only in gene × gene interactions, by analyzing NS subscales or by cross-referencing different personality questionnaires (Salo et al. 2010; Chen et al. 2011; Montag et al. 2012). Because our a priori hypothesis was based on a relatively small sample and to avoid losing power we did not perform any of the extra analyses just mentioned. However, as hypothesized no effects on the temperament dimension HA were observed nor on any of the other TCI scales with the exception of the character scale cooperativeness.

Indeed, as predicted we found that healthy females carrying the Val^158^ homozygote variant scored significantly higher on CO when compared to Met^158^ homozygotes. These findings support the assumption that the Val^158^Met gene influences character traits and not only temperament. Given the higher scores on CO, our results add to the notion that Val^158^ homozygotes are considered to be helpful and empathic, socially tolerant, and compassionate. Indeed, the character scale cooperativeness is based on the concept of self as an integral part of humanity or society; with feelings of community, compassion, conscience, and charity (Kose 2003), in essence, these are all empathic processes. As mentioned earlier, homozygous 9/9VNTR DAT genotypes (higher dopamine levels) display the lowest scores on cooperativeness and compassion (Pełka-Wysiecka et al. 2012). Further, CC genotype carriers – associated with higher dopamine beta-hydroxylase (DBH) activity resulting in higher dopamine turnover to norepinephrine – manifest greater empathic ability compared to CT/TT genotypes (Gong et al. in press). Notwithstanding that for the latter, in addition to lower dopamine levels, empathy-related behaviors may also be determined by the noradrenergic system, these findings link lower dopaminergic activity to a genetic basis of prosocial behaviors (Ebstein et al. 2010). Because homozygote Val^158^ carriers display higher enzymatic activity resulting in less prefrontal dopamine (for the Met^158^ variant this is the reverse) (Heinz and Smolka 2006), our results further support the assumption that Val^158^ allele carriers display higher levels of social facilitation and cooperation and may have the tendency to be more altruistic than Met^158^ homozygotes (Reuter et al. 2011; Walter et al. 2011).

Although all our university students were free from any neuropsychiatric illness, females with only the Met^158^ allele variant scored significant lower on CO compared to Val^158^ homozygotes. Lower scores on CO have been related to more hostile and aggressive behavior (Svrakic and Cloninger 2010). Indeed, Met^158^ homozygotes seem to be more susceptible to emotional difficulties with higher levels of aggression (Albaugh et al. 2010), apathy (Mitaki et al. 2013) and also exhibit greater anxiety (Montag et al. 2008). Furthermore, such individuals are at higher risk to develop mental illnesses (Hosák 2007; Kocabas et al. 2010; Witte and Flöel 2012; Baumann et al. 2013). Men with this Met^158^ variant were found to be more at risk for depression, displayed lower motivational levels and this risk increased in combination with a problematic childhood (Åberg et al. 2011). In another study the trait-anger was found to be significantly associated with both low cooperativeness and depression (Balsamo 2013). Additionally, women with eating disorders carrying the Met^158^ allele variant scored lower on CO than the healthy control group (Mikołajczyk et al. 2010). Of note, in schizophrenic patients having the Met^158^ allele of the COMT gene confers a significantly increased risk for aggressive and violent behavior (Bhakta et al. 2012). In short, our findings support the hypothesis that healthy female homozygote carriers of the COMT Val^158^Met polymorphism (rs4680) are characterized differently on cooperativeness.

However, as the sample is relatively small, the interpretation of these results should be done cautiously. Although the selection of psychopathology-free female subjects within a narrow age range can be considered as a major advantage of the study, by including only healthy women within a certain age range, we cannot generalize our findings to men, older women or individuals with any form of psychiatric illness. Because we did not a priori select our participants based on their genetic COMT Val^158^Met profile, the three groups were unbalanced, which might have influenced our results.

In conclusion, Val^158^ homozygotes differed significantly in cooperativeness when compared to Met^158^ homozygote carriers, indicating that genetics may also play a key role in the development and expression of character features, in our case cooperativeness. A careful selection of individuals may facilitate the detection of COMT Val^158^Met gene influences on distinct aspects of character. Further research is needed to elucidate that more empathic high-scoring CO Val^158^ carriers and more hostile low CO scorers carrying the Met^158^ allele variant are indeed under dopaminergic regulation.
